# Hidden in plain sight: personal factors in autism and ADHD – a protocol for extending the ICF Core Sets for autism and ADHD

**DOI:** 10.3389/fresc.2026.1813836

**Published:** 2026-06-16

**Authors:** Jacintha Tieskens, Melissa H. Black, Emma Asp, Karl Lundin Remnélius, Lovisa Alehagen, John Hasslinger, Johanna Ristolainen Spak, Sven Bölte

**Affiliations:** 1 Center of Neurodevelopmental Disorders (KIND), Department of Women’s and Children’s Health, Centre for Psychiatry Research, Karolinska Institutet & Region Stockholm, Stockholm, Sweden; 2 Department of Community and Clinical Health, School of Allied Health, Human Services & Sport, La Trobe University, Melbourne, VIC, Australia; 3 Child and Adolescent Psychiatry, Stockholm Health Care Services, Stockholm, Sweden; 4 Curtin Autism Research Group, Curtin School of Allied Health, Curtin University, Perth, WA, Australia

**Keywords:** ADHD, autism, ICF, personal factor, person-centered

## Abstract

Autism and ADHD, two of the most common neurodevelopmental conditions, are defined by challenges in daily functioning but are also associated with strengths and can vary substantially in their manifestation across individuals. Yet, diagnostic criteria alone fail to capture their full complexity, particularly on the individual level. A holistic understanding of functioning requires moving beyond defining clinical features to consider the interaction between individual functioning and contextual influences. The International Classification of Functioning, Disability, and Health (ICF) provides a biopsychosocial framework where functioning is understood as the dynamic interplay between the individual's (dis)abilities and the contexts in which they occur. Previously, the ICF has been adapted for tailored use in autism and ADHD using Core Sets, but an important contextual component—personal factors—has been neglected. For a truly holistic understanding of autism and ADHD, including taking into account community perspectives, these personal factors cannot be disregarded. This protocol describes the multi-phase process that will be undertaken to identify and develop shortlists of personal factors most relevant to functioning in individuals diagnosed with autism and ADHD. These shortlists of personal factors will be added to the existing ICF CoreSets platform for autism and ADHD to support a more comprehensive and person-centered approach to autism and ADHD.

## Background

Autism and Attention Deficit Hyperactivity Disorder (ADHD) are two common neurodevelopmental conditions (NDC's) (1, 2). Both are understood as arising from differences in early brain development that influence an individuals' cognitive capacities to comply with societal expectations and demands, leading to educational, occupational, and social challenges (3, 4). Clinical and social services, as well as schools and workplaces, are facing increasing demands to meet the needs of these populations and provide necessary supports (5–7). In the vast majority of service contexts, support for individuals diagnosed with autism and ADHD is primarily guided by diagnostic classification systems, such as the International Classification of Diseases [ICD-11; (4)] and the Diagnostic and Statistical Manual of Mental Disorders [DSM-5-TR; (3)]. While these diagnostic labels have advantages and can provide a useful starting point for understanding certain patterns of behaviour and functioning, an overreliance on diagnostic labels risks marginalizing individuals to a set of criteria and symptoms. Both autism and ADHD are characterized by substantial heterogeneity in how they manifest across individuals (8). Individuals with the same diagnosis may have different lived experiences of the condition, shaped by their unique personal characteristics, life circumstances, co-occurring challenges, and interactions with their environment. A narrow focus on symptoms can obscure the broader contextual factors that influence everyday functioning, making it difficult to understand the specific challenges an individual faces and what support would be most meaningful to them. As a result, care systems often struggle to capture the complexity and individuality of people's lives, leading to fragmented communication, misaligned interventions, and missed opportunities for meaningful support (9).

The neurodiversity paradigm is increasingly embraced by the community and offers an alternative perspective to traditional medical deficit-focused models, viewing autism and ADHD as natural variations of human cognition and behaviour, rather than disorders to be cured (10). It reframes the phenotypes from pathology to variation, emphasizing that the challenges neurodivergent individuals face are often the result of mismatches between individual needs and their environments, rather than merely inherent deficits. This approach advocates for support systems that are tailored to individual strengths, needs, and preferences. Although the neurodiversity paradigm, as well as the urgency of adopting a holistic view of individuals within their unique contexts, is increasingly embraced in practice, the structural dominance of ICD and DSM-based frameworks in healthcare and support services continues to constrain the integration of more comprehensive, person-centered approaches into daily clinical practice (9, 11–13).

**Figure 1:**
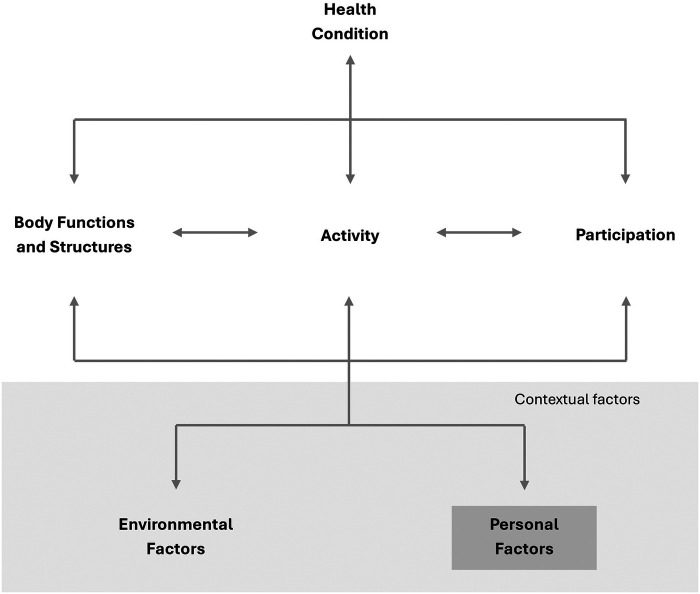
The biopsychosocial framework of the ICF and the position of the personal factors component.

**Figure 2:**
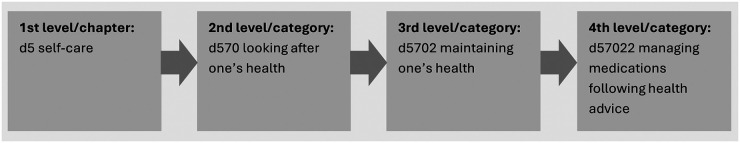
Example of the hierarchically organized category structure of the ICF in the participation component.

Recognizing the multifaceted nature of autism and ADHD is essential for developing tools, supports, and frameworks that are grounded in lived experience. The World Health Organization's (WHO) International Classification of Functioning, Disability and Health [ICF; (14)] offers such a holistic framework. The ICF is based on a biopsychosocial model (15), focusing on an individuals' functioning and disability in context, rather than on symptoms and diagnosis. It conceptualizes functioning as the dynamic interaction between body functions (i.e., physiological and mental functions of the body system), body structures (i.e., anatomical parts of the body), activities (i.e., execution of tasks), participation (i.e., involvement in life situations), and contextual factors (i.e., the conditions or circumstances that shape how an individual interacts with the world) (see Figure 1). The most comprehensive version of the ICF, the *Children and Youth* (ICF-CY) version, presents a total of 1,685 codes with graded levels of increasing detail (level 1–4; see Figure 2 for an example code structure) to operationalize the components of functioning and disability. It comprises 531 codes for body functions, 329 for body structures, 552 for activities and participation, and 273 for environmental factors. The biopsychosocial lens of the ICF allows for a more nuanced understanding of an individual's strengths and challenges, and the supports they may need, given their contextual circumstances (both personal and environmental). Rather than categorizing individuals based on impairments, the ICF emphasizes contextualized functioning, making it a powerful tool for guiding assessment and intervention selection, while at the same time, providing a taxonomy to systematically categorize functioning. Moreover, the ICF provides a shared language that bridges sectors such as health care, education, social services, and policy, facilitating interdisciplinary collaboration and more coherent support systems, which is essential for supporting individuals diagnosed with autism and ADHD (16).

To facilitate the use of the ICF in practice, ICF Core Sets for autism and ADHD have been developed by our team in collaboration with WHO and the ICF Research Branch (17, 18). ICF Core Sets are a selection of ICF categories that are the most relevant for describing functioning in individuals in specific contexts or with specific health conditions (19). Generally, Core Sets aim to include the minimum number of codes required, while still capturing all aspects necessary to describe functioning in individuals with a specific condition across the lifespan, regardless of sex or other potential moderators or mediators. Core Sets are developed through a comprehensive, multi-perspective research process. Since their publication, the autism and ADHD Core Sets have been validated and updated (20, 21), and operationalized and implemented into an online digital platform, the ICF CoreSets platform (22, 23). The Core Sets are increasingly applied in both research and practice (24). Continued feedback from stakeholders, including individuals diagnosed with autism and ADHD, suggests that the Core Sets' comprehensive coverage of functioning domains, their ability to capture strengths and environmental impacts, and their recognition of individual uniqueness, irrespective of diagnosis, are valuable benefits of the Core Sets. However, feedback from stakeholders, along with scientific evidence and practical experience, also suggests that the Core Sets could be further improved to more fully reflect the complexity of functioning in autism and ADHD by adding factors such as personal desire, one's preferred lifestyle, or values (22), which could potentially be covered by the personal factor component of the ICF.

## Personal factors

Within the ICF framework, personal factors are acknowledged as an essential contextual component influencing an individual's functioning, alongside environmental factors (see Figure 1). The WHO states that personal factors capture:

“The particular background of an individual’s life and living, and comprise features of the individual that are not part of a health condition or health states. These factors may include gender, race, age, other health conditions, fitness, lifestyle, habits, upbringing, coping styles, social background, education, profession, past and current experience (past life events and concurrent events), overall behavior patterns and character style, individual psychological assets and other characteristics all or any of which may play a role in disability at any level” [(14), p. 15].

There is broad consensus among researchers that personal factors are essential for understanding functioning, and that their consideration is fundamental to the biopsychosocial model underlying the ICF (25). Despite their recognized importance, personal factors are not classified within the ICF due to large societal and cultural variance and a lack of clarity on the scope of these factors. They are therefore rarely systematically used and not currently captured in the Core Sets for autism and ADHD. Omission of personal factors in these Core Sets, however, limits the ability to fully capture the individuality and complexity of functioning, particularly in conditions such as autism and ADHD, where personal context plays a critical role. For instance, previous research has demonstrated that factors such as gender (26), self-worth (27, 28), feeling of social acceptance (29), and illness belief (30) significantly influence functional outcomes in individuals diagnosed with autism and ADHD.

Although personal factors are not yet formally classified within the ICF, the WHO recognizes their importance and has identified the development of a Personal Factor component as a future priority (14). However, the necessity of developing a taxonomy for personal factors has been debated (31) and several ethical concerns have been raised. One concern is that classifying personal factors could be misused as a classification of the individuals themselves which is inconsistent with the ICF's underlying philosophy of classifying functioning rather than people. Additionally, it has been argued that assigning negative values to personal factors carries a risk of “blaming the victim”. In contrast, others have explored various conceptual approaches and proposed classification systems aimed at defining and structuring personal factors in a systematic and ethically informed way. A variety of conceptualizations and taxonomies for reporting personal factors have been proposed in diverse fields, including audiological rehabilitation (32), motor neuron disease (33), workplace settings (34), and social medicine and rehabilitation (35, 36). See Table 1 for an overview of the known proposed personal factor structures. Although the categorizations greatly differ in regard to their background, and include theoretical approaches from different professional fields, they cover broadly similar content areas. Sociodemographic factors, behavioural patterns, lifestyle, and coping are commonly addressed (37). One particular area where there seems to be variability across conceptualizations and categorizations of personal factors is in relation to subjectivity, and there is no broad consensus in this respect. On one hand, the ICF itself does not preclude entirely the examination of the subjective dimensions of functioning. The ICF linking rules developed by the WHO and ICF Research Branch include perspectives of appraisal (38), considering the extent to which personal expectations and hopes have been achieved (e.g., satisfaction with sleep). On the other, the most frequently encountered perspectives captured by the ICF are descriptive (capturing the extent of the problem) or needs-based (the level of need/support required by an individual), which are ultimately more objective in nature. At the same time, many more subjective dimensions including general life satisfaction, beliefs, values, or preferences remain outside its scope. While there is increasing consensus that subjective information is essential for a comprehensive understanding of functioning, opinions diverge on whether this belongs within the personal factors domain (35, 39–42). Ueda and Okawa (41) emphasize the importance of the subjective dimension in the ICF but argue for a clear distinction, suggesting that personal factors are primarily objective contextual background variables (such as gender, age, social background, and lifestyle), whereas subjective experiences are intrinsic to functioning and disability and belong to a separate dimension. In contrast, Huber and colleagues (39) propose that personal factors should include subjective perceptions, including health-related quality of life, and highlight the reciprocal interactions between ICF components arguing that this way subjectivity is integrated within the whole framework. Also, in Geyh's conceptualization of personal factors, the subjective dimension is explicitly recognized; here personal factors are understood as reflecting the internal context of functioning in terms of individual facts, immediate subjective experience, and recurrent patterns of experience and behaviour (35).

**Table 1:** Overview of previously suggested conceptualizations and structures for ICF's personal factors.

Author and year	Field	Personal factor categorization
Geyh et al. (35)	General (Spinal Cord Injury)	Socio-demographical factors Position in the immediate social and physical context Personal history and biography Feelings Thoughts and beliefs Motives General patterns of experience and behavior
Grotkamp et al. (36)	Social Medicine and prevention	General personal characteristic Physical factors Mental fac tors Attitude Action related skills Behavioral patterns Life situation and socio-eco nomic/-cultural factors.
Heerkens et al. (34)	Work participation	Sociodemographic factors/general personal data (facts) Position in immediate social and physical context Personal history and biography General “mental” personal factors/psychological assets Disease-related factors Lifestyle (habits) Work-related personal factors
Ng and Khan (33)	Motor neuron disease	Demographic factors Emotional states Coping strategies Personality Beliefs Attitudes Others
Salvador-Carulla et al. (54)*	Health-related behavior	Diet and exercise Vitality and stress Sleep Cognition Substance use and Other health risk habits
Stephens and Kerr (32)	Audiological rehabilitation	Personal and demographic characteristics Cognitive factors Behavioral responses Emotions General coping

* Focuses specifically on the categorization of health-related habits (belonging to ICF's personal factor).

Based on the conceptualization of Geyh, Tsuda (43), describes theoretical frameworks that could help identify personal factors and explains how the emotional and motivational aspects in goal pursuit could be captured by the personal factors in the field of rehabilitation. In rehabilitation, but also in mental health care, the focus of support is not only on recovering body functions but also on the individual's autonomy and quality of life. To enable better support for individuals in the long term, outside of the treatment context, it would be essential for practitioners to understand the personal and contextual factors and consider them in goal setting and goal pursuit. Recognizing these personal factors is also integral to person-centered care (25), which prioritizes tailoring interventions to individuals' unique needs, values, preferences, and lifestyles (44). By aligning care with these principles, person-centered approaches can foster greater satisfaction, engagement, and adherence to interventions and support, ultimately contributing to improved mental health outcomes (45).

Previous attempts to conceptualize and categorize ICF personal factors have shown some consensus regarding their content. However, the exact scope remains difficult to define and often depends on the health condition being assessed. Personal factors clearly provide essential context for understanding an individual's functioning in daily life. The omission means neglecting half of the contextual component of the ICF, weakening the holistic and biopsychosocial nature of the framework. Alternatively, leaving the identification and documentation of personal factors to the assessor, as currently recommended by the WHO (14), leads to inconsistent and variable assessment, undermining the goal of standardized and universally applicable functional evaluation (46). For this reason, identifying personal factors relevant to functioning in autism and ADHD, based on a structured and scientific approach, and incorporating them into the existing ICF Core Sets, is necessary to facilitate truly holistic functional assessment and support, including taking into account community voices and experiences.

## Current conceptual framework

Given the lack of clarity regarding the exact scope of personal factors within the ICF it is important to establish a clear conceptual framework for how personal factors are interpreted in this project. Personal factors are understood as individual characteristics or backgrounds that influence functioning but are not part of the health condition itself nor classified within other components of the ICF framework (i.e., body structure, body function, activity, participation, or environmental factors). We acknowledge that daily life functioning, including the role of personal factors, influences and overlaps broader domains such as mental health and quality of life. According to the WHO, mental health is defined as a state of well-being that enables individuals to cope with life's stresses, realize their abilities, and contribute to their communities. Quality of life is defined as an individual's perception of their position in life, shaped by cultural context, value systems, and personal goals. In this project, we examine personal factors in relation to daily life, including functioning, mental health, and quality of life, to avoid overlooking personal factors that may be essential for understanding functioning in individuals diagnosed with autism and/or ADHD. At this stage, we adopt a broad scope and do not impose further boundaries on the definition of personal factors and its outcomes. Although the ICF is explicitly transdiagnostic, the present study focuses on identifying personal factors that are relevant in the context of autism and ADHD. These personal factors should not be interpreted as disorder-specific traits, nor as an attempt to define a universal set of personal factors for inclusion in the ICF—a task that lies within the remit of the WHO-Family of International Classifications (FIC) Network. Rather, they are intended to make explicit those personal characteristics that meaningfully shape how individuals with autism and/or ADHD navigate life. By identifying such empirically grounded and practice-relevant personal factors, this study aims to support clinicians and individuals in translating a diagnostic classification into a more nuanced, individualized understanding of functioning and support needs within an ICF-oriented approach.

## Aim

The aim of this project is to develop shortlists of personal factors relevant to functioning in autism and ADHD to be added to the existing autism and ADHD ICF Core Sets (20, 21). With the addition of these shortlists, we aim to enrich the existing Core Sets and provide a standardized method to capture personal factors within functional assessment for autism and ADHD. Moreover, through this, we aim to facilitate a more personalized, holistic, and neurodiversity-affirmative assessment and understanding of autism and ADHD. Since the ICF does not provide or endorse any official codes for personal factors, coding these factors could support the development of a universal language within the ICF framework. Therefore, we will explore whether the personal factors relevant to functioning in autism and ADHD, identified during the preparatory phase, can be linked to existing comprehensive taxonomies, such as those developed by Geyh et al. (35) and Grotkamp et al. (36). Specific objectives include: 1) identifying personal factors relevant to functioning in people diagnosed with autism and/or ADHD from multiple perspectives (research, professionals, lived experience, and real-life domains); 2) refining the personal factors identified in the first objective to develop shortlists of personal factors that are important to capture in the functional assessment of autism and ADHD using a Delphi process; and 3) integrating the personal factors into the existing autism and ADHD ICF CoreSets platform. The objective of this paper is to outline the process for developing personal factor shortlists for autism and ADHD.

## Method

**Figure 3:**
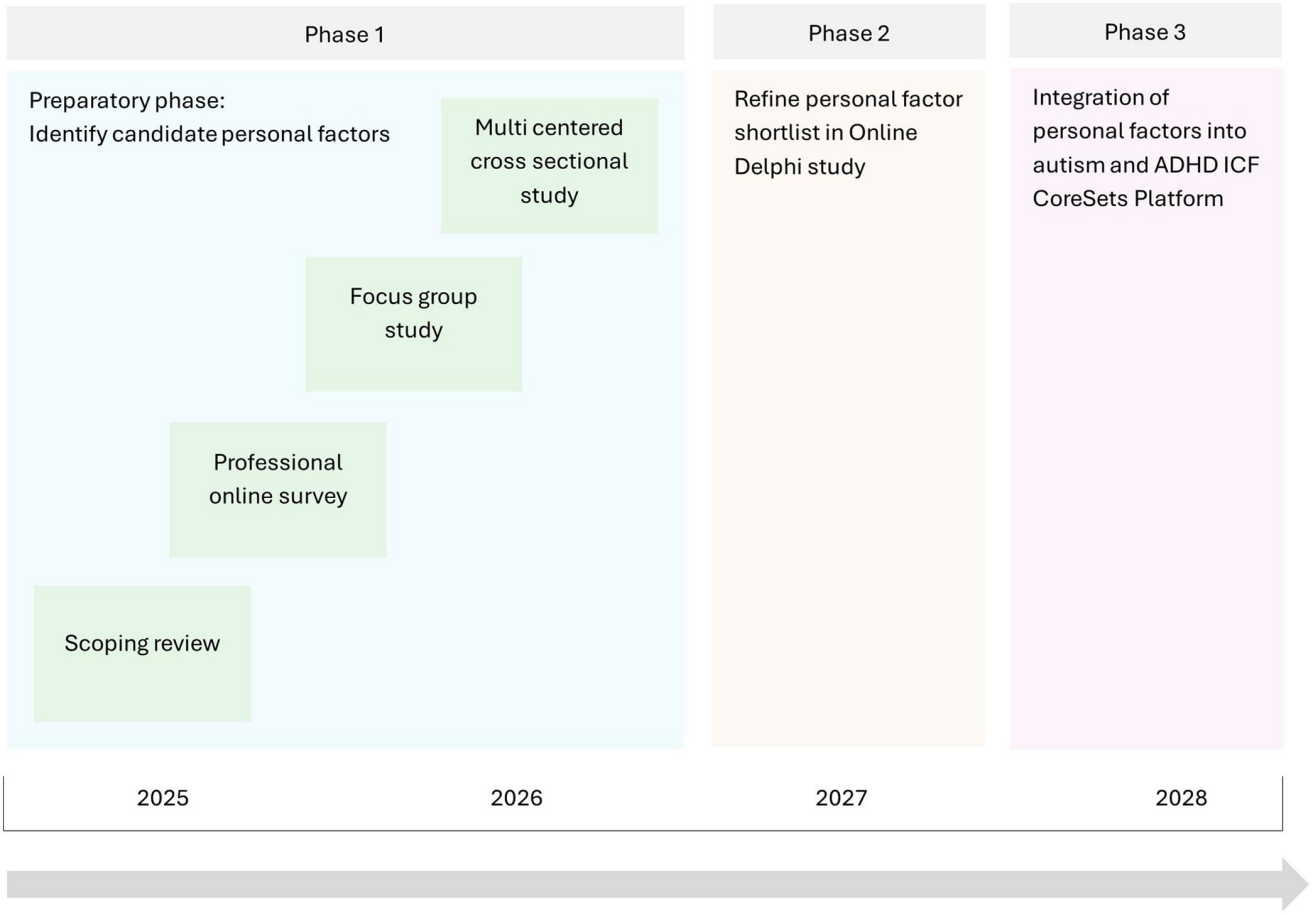
Overview of the project plan.

This project will follow the rigorous multi-phase process established by the WHO and ICF Research Branch (19) which was also used to develop the full autism and ADHD Core Sets (47, 48) (see Figure 3). The first phase consists of four preparatory studies conducted to identify personal factors relevant to functioning in individuals diagnosed with autism and/ or ADHD, capturing perspectives from multiple stakeholders (including research-, professional-, lived experience-, and the real-life domain perspectives) across the six WHO regions globally (African, the Americas, South-East Asia, European, Eastern Mediterranean, Western Pacific). The results of the preparatory studies will be used to develop candidate lists of personal factor codes. The findings of these four studies are combined to integrate complementary perspectives and thereby minimizing perspective-bias in the identification of personal factors. Subsequently, in the second phase, the candidate lists of personal factor codes will be presented during an international online Delphi process to create refined shortlists of personal factors most relevant to functioning in individuals diagnosed with autism and ADHD. The chosen methodology will ensure international perspectives from multiple stakeholders are captured, enabling the use of the newly expanded Core Sets across different cultures and contexts. Finally, in the third phase, the personal factors will be integrated into the existing Core Sets and the ICF CoreSets platform for autism and ADHD. A reference group of individuals diagnosed with autism and/or ADHD will be consulted throughout the project to ensure relevance and appropriateness to the community. See Table 2 for an overview of the project's aims, designs, participants and analyses. The project will be conducted in accordance with the rules and regulations of the Swedish Ethical Review Authority, from which approval has been obtained (2025-04955-01-871193).

**Table 2:** Overview of the project's aims, designs, participants, and analyses.

Phase	Study	Aim	Design	Participants	Analysis
Preparatory phase I (a)	Scoping review	To identify personal factors relevant to autism and ADHD reported in the scientific literature	Scoping review with narrative synthesis	Published studies on personal factors in autism and/or ADHD	Extraction and thematic clustering of personal factor concepts; linking to existing personal factors classification frameworks; frequency counts per personal factor
Preparatory phase I (b)	Survey with professionals	To identify personal factors considered clinically relevant by professionals working with individuals with autism and/or ADHD	Cross-sectional online survey	Clinicians and researchers with expertise in autism and/or ADHD	Thematic analysis of endorsed personal factors; linking to existing personal factor classification frameworks; frequency counts per personal factor
Preparatory phase I (c)	Qualitative study	To identify personal factors considered relevant from the perspective of individuals with autism and/or ADHD or their relatives	Qualitative design using focus groups and interviews	Individuals with autism and/or ADHD (> 7 years) or their relatives (> 18 years)	Thematic analysis of endorsed personal factors; linking to existing personal factor classification frameworks; frequency counts per personal factor
Preparatory phase I (d)	Cross-sectional multicenter study	To identify personal factors relevant in real life domains	Analysis of relevant personal factors in real-life domains	Individuals with autism and/or ADHD and/or their service provider	Conceptual analysis of the endorsed personal factors; linking to existing personal factor classification frameworks; frequency counts per personal factor
Consensus phase II	International Online Delphi Study	To reach consensus on the inclusion and clustering of personal factors	Multi-round Delphi study	International multidisciplinary expert panel including clinicians, researchers, and people with lived experience	Round-by-round descriptive analysis (using CREDES guidelines); consensus will be determined using predefined criteria (e.g., percentage agreement or statistical thresholds)
Integration phase III	Integration of the personal factor shortlist into the ICF Core Sets Platform for autism and ADHD	To operationalize the personal factors into clinical usable items, that can be integrated in the ICF Core Set platform	Operationalization of personal factors	International multidisciplinary expert panel including clinicians, researchers, and people with lived experience	An iterative process will be undertaken, using common questionnaire design principles

### Phase One

#### Preparatory study one: scoping review (research perspective)

A scoping review will be conducted to capture the personal factors that have been associated with functioning and disability in autism and/or ADHD in the literature. The objectives are to (1) identify which personal factors are investigated within the context of daily life functioning in autism and ADHD, and (2) link the personal factors to an existing taxonomy developed in previous work (35, 36) in order to quantify the relevance of the personal factors according to scientific literature. A comprehensive database search will identify articles investigating personal factors that may influence functioning in autism and ADHD using a search strategy developed in consultation with experienced university librarians at Karolinska Institutet. The systematic literature search will be conducted in Medline/PubMed, PsychINFO, Web of Science, ERIC, and CINAHL. Search terms will be organized according to the target group (i.e., autism and ADHD), personal factors (e.g., attitude, lifestyle, value, belief), and outcome (e.g., functioning, wellbeing, quality of life). Included articles will date back no longer than 10 years. Articles identified through the search process will be screened for eligibility at the level of title and abstract. Filtered articles will then undergo screening at a full-text level to determine inclusion. Data synthesis will be undertaken in two phases: First, a narrative synthesis will be undertaken by grouping articles and findings thematically. Second, identified personal factors will be linked to an existing taxonomy developed in previous work (35, 36). Although personal factors fall outside scope of the ICF, our linking process will follow established linking rules for linking content to the ICF (38) and procedures for linking personal factors that have been employed previously (49). To enhance the credibility and dependability of the analysis, two researchers who have completed training from the WHO ICF Research Branch, will independently link the identified personal factors to the existing personal factor coding systems. Disagreements will be discussed until consensus is achieved. Where disagreement cannot be resolved a third, or fourth researcher from the team, also experienced with the ICF and training from the WHO ICF Research branch, will be consulted. Absolute and relative frequencies of personal factor concepts and codes to which the concepts are linked will be reported. In accordance with the guidelines, a category will be counted once for each study (19).

#### Preparatory study two: survey with professionals (opinion leader perspective)

An international online survey will be conducted among professionals in the field of autism and ADHD to gather their perspectives on the personal factors they believe are most relevant to functioning in autism and/or ADHD. Participants will include professionals (e.g., researchers, medical doctors, psychologists, social workers, educators, occupational therapists, and other professionals with relevant experience) with over five years of experience working with individuals (or relatives of individuals) diagnosed with autism and/or ADHD. An internationally diverse sample with varied work experience will be sought. Recruitment will occur through professional networks and organizations, with efforts made to recruit professionals across all six WHO regions. In addition, a web-based search will be conducted by two researchers to identify collaborators, expert networks in autism and/or ADHD, and clinicians with professional experience working with individuals with autism and/or ADHD. Eligible professionals will be asked to recommend further possible participants. Professionals will receive an online survey with a brief explanation of the ICF and personal factors, followed by open-ended questions on which personal factors they consider relevant for functioning in autism and/or ADHD. The survey includes questions based on those proposed in guidelines for the development of ICF Core Sets (19), but adapted for our purpose, such as “Based on your experience, which *personal factor(s)* do you consider important to assess in order to gain a better understanding of daily life functioning and support needs in people with a diagnosis of autism and/or ADHD?” The survey will also include questions assessing whether identified personal factors are perceived as specific to particular diagnostic groups, age groups, or genders. Survey information and questions will be formulated to ensure that the broad variety of possible personal factors are covered and will be informed, in part, by findings from the previously performed scoping review. The survey will be reviewed by the reference group prior to administration and piloted to evaluate clarity, comprehensibility, and feasibility before full implementation. Data-collection will continue until saturation is achieved. Saturation will be considered reached when no new personal factors or relevant insights emerge from at least ten consecutive surveys. Based on prior surveys conducted with the aim of identifying ICF Core Set and shortlists, we anticipate that this may occur at approximately 200 professionals. Collected data will be analyzed in accordance with the established guidelines for two-phase analysis of qualitative data using the ICF (38). Phase one comprises an inductive qualitative content analysis approach to extract meaningful concepts. The second, deductive, phase links these meaningful concepts to an existing taxonomy developed in previous work (35, 36) according to the ICF linking rules (38). The same procedure will be used as described in the previous preparatory study (scoping review). Absolute and relative frequencies of personal factor concepts and codes to which the concepts are linked will be reported. In accordance with the guidelines, a category will be counted once for each expert (19). In addition, a descriptive analysis will be conducted to explore whether different personal factors are identified across diagnosis, age and gender or other demographic background variables.

#### Preparatory study three: qualitative study (lived-experience perspective)

A qualitative focus group/interview study will be conducted to identify personal factors relevant to functioning from the perspective of people diagnosed with autism and ADHD themselves, and those closest to them (i.e., caregivers and those involved in their daily life). Participants will include people with a diagnosis of autism and/or ADHD aged 7 years and older with the ability to communicate their experiences using spoken language or other communication like written or augmentative and alternative communication (AAC), as well as individuals over 18 years who are caregivers or people involved in the daily lives of individuals with a diagnosis. Participants from all six WHO regions will be sought across ages (children to older adults), sex/gender, and ethnicity. Participants will not be excluded on the basis of co-occurring psychiatric diagnoses reflecting the high rates of co-occurrence in autism and ADHD and enhancing the ecological validity of the sample. In addition, there are no restrictions on developmental age, as long as the participant is able to communicate their experiences, with or without AAC. Recruitment will occur through our own network interest organizations, social media, clinics, and other established channels of the research team. Data collection will continue until saturation is achieved. Saturation will be considered reached when no new information is identified from at least two consecutive focus groups or interviews. Data will be collected through focus group interviews consisting of 4–8 participants. Each focus group will begin with a brief explanation of the ICF framework and personal factors, followed by open-ended questions exploring which personal factors participants perceive as influencing daily life functioning. Alternatively, one-on-one interviews are offered to accommodate individual preferences and logistical issues. For younger participants, or for those who prefer to participate in the interview together with a trusted person, this option is provided. Interviews are conducted either in person, over the phone, or online (e.g., via Zoom or similar platforms), and will be moderated by experienced researchers and clinicians. The interview questions are to be developed in consultation with the reference group. They will be informed by the preceding preparatory studies and will be formulated to ensure the broad variety of possible personal factors are covered. In addition, an online questionnaire on demographic information will be assessed. The interview questions will also be piloted to assess clarity, comprehensibility, and feasibility prior to full deployment. Data will be analyzed using the same two-phase process described in the professional survey study. Absolute and relative frequencies of personal factor concepts and categories to which the concepts are linked will be reported. In accordance with the guidelines, a category will be counted once for each participant (19). In addition, a descriptive analysis will be conducted to explore whether different personal factors are identified across diagnosis, age and gender or other demographic background variables.

#### Preparatory study four: cross-sectional multicenter study (real-life perspective)

An empirical cross-sectional study will identify the personal factors that are relevant for functioning in individuals diagnosed with autism and/ or ADHD in real-life domains. Participants will include individuals with a diagnosis of autism and/or ADHD, who attend clinical centers, education or employment, social services, and leisure settings (e.g., sport clubs) across the six WHO regions. Approximately 100 participants will be recruited through our own networks interest organizations, social media, clinics, and other established channels of the research team. Professionals in different settings will be asked to identify eligible clients. When the client consents, the professional is asked to complete a personal factor checklist developed based on the preceding studies, following a similar structure to the ICF checklist (50). This questionnaire will also collect demographic information. The checklist will include a combination of health records, documentation, observations, and semi-structured interviews assessed by professionals. Alternatively, the checklist can be completed by the participants themselves (for 7-year-olds and older) or with help from a researcher. Descriptive statistics will be provided, and absolute and relative frequencies of personal factor concepts and codes will be reported. See the previous preparatory studies for a description of the linking process. In addition, a descriptive analysis will be conducted to explore whether different personal factors are identified across diagnosis, age and gender or other demographic background variables.

#### Phase two

Following the completion of the four preparatory studies, all identified personal factors will be systematically integrated into a single candidate list by the research team. Personal factors identified across studies will be pooled, deduplicated, and assigned a provenance profile indicating in which preparatory studies they were identified, how frequently they were mentioned within each study, and how they link to the existing personal factor classification frameworks (35, 36). The candidate list will be organized into thematic clusters based on conceptual similarity. This structured candidate list, with cluster organization and provenance profiles visible to participants, will serve as the input for the Delphi process.

#### International online delphi study

Results from the preparatory studies will be further processed during an international online Delphi process to identify the most relevant personal factors for functioning in individuals with autism and/or ADHD. Participants will be approximately 30 experts with clinical, academic, and/or lived expertise relevant to autism and/or ADHD. Experts are individuals with lived experience of autism and/or ADHD or their relatives and professionals (e.g., researchers, medical doctors, psychologists, social workers, educators, occupational therapists, and other professionals with relevant experience) with over five years of experience working with individuals (or relatives of individuals) diagnosed with autism and/or ADHD. Participants will be eligible if they are aged 18 years or older and can read and respond to online survey materials in English. The Delphi approach is an iterative, structured method involving multiple rounds of questionnaires and discussions aiming to seek consensus. The Conducting and REporting DElphi Studies (CREDES) guidelines will be used as a framework (51). Up to three rounds will be conducted entirely online. Consensus will be determined using predefined criteria (e.g., percentage agreement or statistical thresholds), specified prior to the first Delphi round, in combination with structured qualitative feedback and iterative discussion across rounds via the online platform. In addition to the personal factor concept, the experts will be presented with several open-ended questions including age-specific and diagnosis-specific considerations for the development of potential separate shortlists. This iterative methodology ensures rigorous refinement of the personal factors, resulting in the development of a final set of personal factor codes necessary to capture when assessing functioning in individuals with autism and/or ADHD.

#### Phase three

#### Integration of the personal factor shortlist into the ICF CoreSets platform for autism and ADHD

The shortlists of personal factors will be operationalized into a set of items for both self- and informant reports. A procedure similar to the one used to operationalize the autism and ADHD Core Set codes into items for the ICF CoreSets platform will be applied (22). An iterative process involving stakeholders and professionals from different countries will be undertaken, using common questionnaire design principles (52).

## Discussion

This protocol paper outlines the process of developing shortlists of personal factors relevant to functioning in individuals diagnosed with autism and/or ADHD, with the aim of integrating them into the ICF Core Sets for these conditions. By incorporating standard lists of personal factors that are considered most important by multiple stakeholders, the Core Sets for autism and ADHD will be expanded to fully reflect the biopsychosocial model underlying the ICF framework. These enriched Core Sets will support more inclusive, context-sensitive, and person-centered approaches in assessment and support planning for individuals diagnosed with autism and/or ADHD.

Incorporating the personal factors in a systematic manner further supports the aim of the ICF Core Sets for autism and ADHD to allow for a more nuanced understanding of functioning, moving beyond diagnostic categories to reflect individuals holistically, including their strengths, challenges, and contexts from multiple perspectives, including lived experience. This will yield several advantages for assessment, intervention planning, as well as for the general approach to individuals with a diagnosis of autism and/or ADHD. First, including personal factors enables a more nuanced understanding of individuals' unique characteristics and strengths. As these factors are, by definition, independent of diagnostic categories, they offer a non-pathologizing perspective that is consistent with the neurodiversity paradigm. Second, although personal factors are often considered during clinical practice, they frequently lack a structured and standardized format for documentation in medical records. Integrating these factors systematically into the ICF Core Sets would support more consistent and systematic reporting, enhancing the quality and completeness of assessments. Finally, rather than viewing functioning solely through the lens of diagnostic categories and functional abilities and disabilities, personal factors allow us to consider the unique traits, preferences, beliefs, values, coping styles, and life experiences that shape how a person engages with their environment. This shift acknowledges that functioning is influenced by dynamic interactions between both internal and external contextual factors. By adding a personal factors shortlist/s, we both adhere to the holistic framework of the ICF as well as to the standardized and universally applicable language that the ICF provides.

### Challenges

Developing systematic categorized shortlists of personal factors relevant to autism and ADHD will not be without challenges. First, the identification of personal factors involves nuanced and sensitive considerations, particularly in the context of autism and ADHD. While the ICF framework clearly defines personal factors as distinct from health conditions and health states to maintain conceptual clarity, this distinction is not always straightforward in practice. In NDC's such as autism and ADHD, the boundaries between what may be considered a personal factor and what is viewed as part of the health condition are often blurred. This complexity is further amplified by the neurodiversity paradigm, which frames autism and ADHD as natural variations in human functioning rather than disorders. Within this perspective, the distinction between personal factors and health condition may not be meaningful or even present. However, a sole reliance on diagnostic frameworks like the ICD or DSM can sometimes lead to personal factors being unintentionally framed as symptoms. For instance, the personal factor of “having strong/deep interests” or “being passionate” may be interpreted as a trait of the condition due to its resemblance to the ICD/DSM criterion: “highly restricted, fixated interests that are abnormal in intensity or focus”. However, explicitly identifying “having strong/deep interests” or “being passionate” as a personal factor encourages clinicians to critically reflect on such traits and consider their relevance to functioning in a more nuanced and individualized way. Additionally, there may be overlap between personal factors and other components of functioning within the ICF framework. For instance, individual psychological assets, by the WHO considered as part of the personal factor, can also be classified under the code for body functions “b126 Temperament and personality functions” (e.g., b 1,265 optimism). Importantly, the aim of this project is not to classify traits on an individual level as either personal factors, body functions or symptoms of a health condition. Instead, our goal is to identify personal factors that are relevant to functioning in individuals diagnosed with autism and/or ADHD, and that are not yet captured by the existing components of the ICF. By doing so, we aim to expand the ICF Core Sets in a way that supports more person-centered assessment and planning. While this approach may involve navigating some ambiguity, it broadens the perspective through which health conditions are assessed.

A second challenge is the absence of a universally accepted classification system for personal factors, particularly within the field of NDC's. As noted in the introduction, personal factors are acknowledged in the ICF framework but remain uncoded, resulting in a lack of standardized documentation and assessment. For the systematic application of the personal factor shortlists, it is essential that identified concepts can be linked to a coherent and universal taxonomy. Among the existing frameworks, the taxonomies developed by Geyh et al. (35) and Grotkamp et al. (36) offer the most comprehensive and structured approaches to categorizing personal factors. Both aim to be applicable across diverse health conditions and settings and seek to clearly distinguish personal factors from other components of the ICF. Geyh's taxonomy is organized into seven chapters in which psychological and experiential dimensions are emphasized, including short-term feelings and subjective aspects of personal factors. Grotkamp's taxonomy, on the other hand, is structured into five chapters that are more oriented toward long-term and stable conditions, with a focus on practical application in rehabilitation and social medicine. Although both taxonomies appear applicable to the field of autism and ADHD, their relevance and suitability must be critically evaluated throughout our research project. These conditions present unique ways in which personal factors are expressed, experienced, and interpreted, and it is crucial that the taxonomy is sensitive to these nuances. In the preparatory studies, we will begin by applying both the taxonomy of Geyh et al. (35) and Grotkamp et al. (36) to structure the identified personal factors. The findings from these studies will guide us in assessing the extent to which each taxonomy captures the complexity and specificity of personal factors in autism and ADHD. This process, together with insights from the Delphi study, will inform our decision on which, if any, taxonomy (or combination there-of) offers the best conceptual and practical fit for this population.

Third, both autism and ADHD persist across the life span, and both someone's functioning and the context in which it occurs may vary in nature with age. This is also reflected in the age-specific Core Sets that have been developed for autism and ADHD (17, 18). Consequently, the personal factors that are most relevant to functioning are also likely to vary across different life stages. Developing a universal shortlist of personal factors applicable to all individuals diagnosed with autism and ADHD, regardless of age, may therefore be challenging. In the preparatory studies, we will explore how personal factors differ across the life course. These studies will provide insight into whether life stage-specific shortlists of personal factors are needed and will be further addressed in the Delphi process.

Fourth, while this project is designed to develop a shortlist of personal factors relevant to functioning in both autism and ADHD, we acknowledge that these conditions, although frequently co-occurring (53), differ in their clinical presentation, lived experiences, and developmental trajectories. It is therefore reasonable to question whether a single list can adequately capture the personal factors pertinent to both groups. However, the ICF framework is intentionally diagnosis-independent and etiologically neutral, which aligns with a transdiagnostic approach. Developing a shared list for autism and ADHD is consistent with this principle and may facilitate a more inclusive understanding of personal factors across NDC's. In the preparatory studies, we will specifically explore how personal factors differ across autism and ADHD. These studies will provide insight into whether health condition specific shortlists of personal factors are needed, which will be further addressed in the Delphi process.

Fifth, several potential sources of bias should be considered. These include publication bias in the scoping review, perspective-specific biases related to professional, lived-experience, and multicentre inputs, and limited global representation due to language constraints. Additionally, the requirement for certain communication ability with or without AAC in the qualitative sub-study may restrict direct inclusion of individuals with certain intellectual abilities. These biases will be addressed through the integration of findings across the preparatory studies, transparent reporting across sub-studies and the Delphi process, which will aim to include a diverse expert panel. In addition, to mitigate the restricted inclusion of individuals with specific intellectual abilities, we also include relatives of individuals with autism and/or ADHD to capture perspectives from individuals with varying intellectual abilities.

Last is the critical and often debated issue of whether personal factors need to be classified in the same way as the other components of the ICF and the risk that such coding may be perceived as attributing responsibility of the disability to the individual or the risk of “blaming the victim” (31, 46). We fully acknowledge the ethical sensitivity of this concern and consider it a central point of attention in our project. However, the potential for misinterpretation underscores the importance of a careful and reflective approach to conceptualizing and operationalizing personal factors. Rather than solely assessing an individual's functioning and disability within their external environment, the formal integration of personal factor codes into the ICF Core Sets for autism and ADHD can be viewed as an opportunity to represent the person in a more holistic manner, taking into account their personal background, characteristics, and attributes. This includes not only the challenges an individual may face, but also their strengths and resources. By developing the personal factor shortlist in a systematic way, guided by a reference group and based on input from relevant stakeholders, we aim to support a more individualized and needs-based approach to care and support, which will mitigate the potential misuse of personal factors. At the same time, this requires a shared responsibility among researchers, clinicians, and policymakers to ensure that personal factors are described and applied in a respectful, non-stigmatizing, and empowering manner.

## Conclusion

This project will further work towards a more holistic approach to person-centered assessment of functioning and support by developing a personal factor shortlist to be incorporated into the existing ICF Core Sets for autism and ADHD. Incorporating personal factors aligns with priorities of the NDC community and their wishes for a more strengths-based and person-centered approach towards assessment of functioning. Relevance and accuracy of the personal factor codes will be ensured through the multi-phased, scientific process of development, where the six WHO regions are represented across clinical, research, and real-life domains. This project marks an important shift in focus and provides significant updates to the Core Sets for autism and ADHD.

## Data Availability

The original contributions presented in the study are included in the article/Supplementary Material, further inquiries can be directed to the corresponding author.

## References

[1] Popit S Serod K Locatelli I Stuhec M. Prevalence of attention-deficit hyperactivity disorder (ADHD): systematic review and meta-analysis. Eur Psychiatry. (2024) 67:e68. 10.1192/j.eurpsy.2024.1786 39381949 PMC11536208

[2] Zeidan J Fombonne E Scorah J Ibrahim A Durkin MS Saxena S. Global prevalence of autism: a systematic review update. Autism Res. (2022) 15:778–90. 10.1002/aur.2696 35238171 PMC9310578

[3] American Psychiatric Association. Diagnostic and Statistical Manual of Mental Disorders. 5th ed., text revision (DSM-5-TR) ed. Washington, DC: American Psychiatric Association (2022).

[4] World Health Organization. International Classification of Diseases Eleventh Revision (ICD-11). Geneva: World Health Organization (2022).

[5] Coghill D Banaschewski T Cortese S Asherson P Brandeis D Buitelaar J. The management of ADHD in children and adolescents: bringing evidence to the clinic: perspective from the European ADHD guidelines group (EAGG). Eur Child Adolesc Psychiatry. (2023) 32:1337–61. 10.1007/s00787-021-01871-x 34677682 PMC8532460

[6] Mandy W. Six Ideas About how to Address the Autism Mental Health Crisis. London, England: SAGE Publications Sage UK (2022). p. 289–92. 10.1177/13623613211067928 35109701

[7] Hobbs T, Berry V, Fonagy P. Editorial perspective: a systems approach to addressing young people's mental health. *J. Child Psychol. Psychiatry.* (2025) 66:271–74. doi: 10.1111/jcpp.14077 PMC11754711 39586677

[8] Thapar A Cooper M Rutter M. Neurodevelopmental disorders. Lancet Psychiatry. (2017) 4:339–46. 10.1016/S2215-0366(16)30376-5 27979720

[9] Bölte S. A more holistic approach to autism using the international classification of functioning: the why, what, and how of functioning. Autism. (2023) 27:3–6. 10.1177/13623613221136444 36330803

[10] Den Houting J. Neurodiversity: An Insider’s Perspective. London, England: Sage Publications Sage UK (2019). p. 271–3. 10.1177/1362361318820762 30556743

[11] van Hulst BM Groen-Blokhuis MM de Ridder B Dekkers TJ. Commentary: are we over-pathologising young people’s mental health? Locked inside our own building–on disorderism and the need to deflate our language. Child Adolesc Ment Health. (2025) 30(4):397–9. 10.1111/camh.70028 40956280 PMC12573061

[12] Timimi S. Debate: are we over-pathologising young people’s mental health? You'd be in denial if you think we aren't. Child Adolesc Ment Health. (2025) 30:296–8. 10.1111/camh.70015 40673361

[13] Batstra L Frances A. Diagnosing the context is as important as diagnosing the individual. Front Psychiatry. (2025) 16:1698878. 10.3389/fpsyt.2025.1698878 41210139 PMC12589039

[14] World Health Organization. International Classification of Functioning, Disability and Health (ICF). Geneva: World Health Organization (2001).

[15] Engel GL. The need for a new medical model: a challenge for biomedicine. Science. (1977) 196:129–35. 10.1126/science.847460 847460

[16] Kristensen RK Andersen PT Bilenberg N Milling ED Dalgaard Guldager J. Mapping the landscape and evidence of cross-sectoral collaboration models targeting individuals referred for assessment of attention-deficit hyperactivity disorder or autism spectrum disorder: protocol for a scoping review. BMJ Open. (2025) 15:e088850. 10.1136/bmjopen-2024-088850 39819944 PMC11751781

[17] Bölte S Mahdi S Coghill D Gau SS-F Granlund M Holtmann M. Standardised assessment of functioning in ADHD: consensus on the ICF core sets for ADHD. Eur Child Adolesc Psychiatry. (2018) 27:1261–81. 10.1007/s00787-018-1119-y 29435654

[18] Bölte S Mahdi S de Vries PJ Granlund M Robison JE Shulman C. The gestalt of functioning in autism spectrum disorder: results of the international conference to develop final consensus international classification of functioning, disability and health core sets. Autism. (2019) 23:449–67. 10.1177/1362361318755522 29378422 PMC6376609

[19] Selb M Escorpizo R Kostanjsek N Stucki G ÜSTüN B Cieza A. A guide on how to develop an international classification of functioning, disability and health core set. Eur J Phys Rehabil Med. (2015) 51:105–17. 24686893

[20] Bölte S Alehagen L Black MH Hasslinger J Wessman E Lundin Remnélius K. The gestalt of functioning in autism revisited: first revision of the international classification of functioning, disability and health core sets. Autism. (2024) 28:2394–411. 10.1177/13623613241228896 38351521 PMC11402269

[21] Bölte S Alehagen L Black MH Hasslinger J Wessman E Remnélius KL. Assessment of functioning in ADHD according to world health organization standards: first revision of the international classification of functioning, disability and health core sets. Dev Med Child Neurol. (2024) 66:1201–14. 10.1111/dmcn.15865 38308443 PMC11579801

[22] Alehagen L Hasslinger J Wessman E Black M Lundin Remnélius K Helander J. Operationalizing the ICF core sets for autism and ADHD: a multiple-methods feasibility study. J Autism Dev Disord. (2025):1–16. 10.1007/s10803-024-06717-4 PMC13222184 39883295

[23] Alehagen L Hasslinger J Black MH Wessman E Bölte S. Psychometric properties of the operationalized ICF core sets for autism and ADHD: item metrics, reliability, and validity. Disabil Rehabil. (2025):1–28. 10.1080/09638288.2025.2581019 41177936

[24] Alehagen L Bölte S Black MH. Application of the international classification of functioning, disability, and health in autism and attention-deficit hyperactivity disorder: a scoping review. Autism Int J Res Pract. (2025) 29:310–28. 10.1177/13623613241272044 PMC11816479 39183470

[25] Karhula M Saukkonen S Xiong E Kinnunen A Heiskanen T Anttila H. ICF Personal factors strengthen commitment to person-centered rehabilitation–A scoping review. Front Rehabil Sci. (2021) 2:709682. 10.3389/fresc.2021.709682 36188794 PMC9397796

[26] Lundin K Mahdi S Isaksson J Bolte S. Functional gender differences in autism: an international, multidisciplinary expert survey using the international classification of functioning, disability, and health model. Autism Int J Res Pract. (2021) 25:1020–35. 10.1177/1362361320975311 33267637

[27] Schei J Nøvik TS Thomsen PH Lydersen S Indredavik MS Jozefiak T. What predicts a good adolescent to adult transition in ADHD? The role of self-reported resilience. J Atten Disord. (2018) 22:547–60. 10.1177/1087054715604362 26399710

[28] Dvorsky MR Langberg JM Becker SP Evans SW. Trajectories of global self-worth in adolescents with ADHD: associations with academic, emotional, and social outcomes. J Clin Child Adolesc Psychol. (2019) 48:765–80. 10.1080/15374416.2018.1443460 29714502 PMC6287970

[29] Ray AR Evans SW Langberg JM. Factors associated with healthy and impaired social functioning in young adolescents with ADHD. J Abnorm Child Psychol. (2017) 45:883–97. 10.1007/s10802-016-0217-x 27796691 PMC5409909

[30] Wong IYT Hawes DJ Dar-Nimrod I. Illness representations among adolescents with attention deficit hyperactivity disorder: associations with quality of life, coping, and treatment adherence. Heliyon. (2019) 5:e02705. 10.1016/j.heliyon.2019.e02705 31687524 PMC6820282

[31] Leonardi M Sykes CR Madden RC ten Napel H Hollenweger J Snyman S. Do we really need to open a classification box on personal factors in ICF? Disabil Rehabil. (2016) 38:1327–8. 10.3109/09638288.2015.1089604 26457794

[32] Stephens D Kerr P. Auditory disablements: an update: discapacidad auditiva: una actualization. Audiology. (2000) 39:322–32. 10.3109/00206090009098013 11766692

[33] Ng L Khan F. Identification of personal factors in motor neurone disease: a pilot study. Rehabil Res Pract. (2011) 2011:871237. 10.1155/2011/871237 22110980 PMC3195318

[34] Heerkens YF de Brouwer CP Engels JA van der Gulden JW Kant I. Elaboration of the contextual factors of the ICF for occupational health care. Work. (2017) 57:187–204. 10.3233/WOR-172546 28582939

[35] Geyh S Schwegler U Peter C Müller R. Representing and organizing information to describe the lived experience of health from a personal factors perspective in the light of the international classification of functioning, disability and health (ICF): a discussion paper. Disabil Rehabil. (2019) 41:1727–38. 10.1080/09638288.2018.1445302 29509044

[36] Grotkamp S Cibis W Brüggemann S Coenen M Gmünder HP Keller K. Personal factors classification revisited: a proposal in the light of the biopsychosocial model of the world health organization (WHO). Aust J Rehabil Counsell. (2020) 26:73–91. 10.1017/jrc.2020.14

[37] Müller R Geyh S. Lessons learned from different approaches towards classifying personal factors. Disabil Rehabil. (2015) 37:430–8. 10.3109/09638288.2014.923527 24856787

[38] Cieza A Fayed N Bickenbach J Prodinger B. Refinements of the ICF linking rules to strengthen their potential for establishing comparability of health information. Disabil Rehabil. (2019) 41:574–83. 10.3109/09638288.2016.1145258 26984720

[39] Huber JG Sillick J Skarakis-Doyle E. Personal perception and personal factors: incorporating health-related quality of life into the international classification of functioning, disability and health. Disabil Rehabil. (2010) 32:1955–65. 10.3109/09638281003797414 20441436

[40] McDougall J Wright V Rosenbaum P. The ICF model of functioning and disability: incorporating quality of life and human development. Dev Neurorehabil. (2010) 13:204–11. 10.3109/17518421003620525 20450470

[41] Ueda S Okawa Y. The subjective dimension of functioning and disability: what is it and what is it for? Disabil Rehabil. (2003) 25:596–601. 10.1080/0963828031000137108 12959333

[42] Salvador-Carulla L Lucas R Ayuso-Mateos JL Miret M. Use of the terms” wellbeing” and” quality of life” in health sciences: a conceptual framework. Eur J Psychiatry. (2014) 28:50–65. 10.4321/S0213-61632014000100005

[43] Tsuda A Manalo E Miyai I Noda T. Efficient integration of personal factors into the international classification of functioning, disability, and health (ICF): the importance of emotional and motivational aspects in goal pursuit. Front Rehabil Sci. (2024) 5:1450157. 10.3389/fresc.2024.1450157 39678126 PMC11638191

[44] Coulter A Oldham J. Person-centred care: what is it and how do we get there? Future Hosp J. (2016) 3:114–6. 10.7861/futurehosp.3-2-114 31098200 PMC6465833

[45] Coulter A Entwistle VA Eccles A Ryan S Shepperd S Perera R. Personalised care planning for adults with chronic or long-term health conditions. Cochrane Database Syst Rev. (2015) 2015:CD010523. 10.1002/14651858.CD010523.pub2 25733495 PMC6486144

[46] Simeonsson RJ Lollar D Björck-Åkesson E Granlund M Brown SC Zhuoying Q. ICF and ICF-CY lessons learned: pandora’s box of personal factors. Disabil Rehabil. (2014) 36:2187–94. 10.3109/09638288.2014.892638 24601863

[47] Bölte S de Schipper E Holtmann M Karande S de Vries PJ Selb M. Development of ICF core sets to standardize assessment of functioning and impairment in ADHD: the path ahead. Eur Child Adolesc Psychiatry. (2014) 23:1139–48. 10.1007/s00787-013-0496-5 24337412 PMC4246121

[48] Bölte S de Schipper E Robison JE Wong VC Selb M Singhal N. Classification of functioning and impairment: the development of ICF core sets for autism spectrum disorder. Autism Res. (2014) 7:167–72. 10.1002/aur.1335 24124074

[49] Black MH Helander J Segers J Ingard C Bervoets J de Puget VG. Resilience in the face of neurodivergence: a scoping review of resilience and factors promoting positive outcomes. Clin Psychol Rev. (2024) 113:102487. 10.1016/j.cpr.2024.102487 39178757

[50] World Health Organization. ICF checklist (2003). Available online at: https://www.who.int/publications/m/item/icf-checklist (Accessed January 12, 2026).

[51] Jünger S Payne SA Brine J Radbruch L Brearley SG. Guidance on conducting and REporting DElphi studies (CREDES) in palliative care: recommendations based on a methodological systematic review. Palliat Med. (2017) 31:684–706. 10.1177/0269216317690685 28190381

[52] Krosnick JA Presser S. Question and questionnaire design. In: Wright JD Marsden PV, editors. Handbook of Survey Research. Bingley: Emerald Group Publishing (2010). p. 264–313.

[53] Hatch B Kadlaskar G Miller M. Diagnosis and treatment of children and adolescents with autism and ADHD. Psychol Sch. (2023) 60:295–311. 10.1002/pits.22808 37065905 PMC10092654

[54] Salvador-Carulla L Alonso F Gomez R Walsh CO Almenara J Ruiz M. Basic concepts in the taxonomy of health-related behaviors, habits and lifestyle. Int J Environ Res Public Health. (2013) 10:1963–76. 10.3390/ijerph10051963 23670578 PMC3709359

